# Emotion regulation and depressive symptoms mediate the association between chronotype and suicidality

**DOI:** 10.1017/neu.2026.10077

**Published:** 2026-04-10

**Authors:** Eunseo Choo, Hangyu Kim, Jakyung Lee, Hyeona Yu, Hyun Jung Hur, Tae Hyon Ha, Woojae Myung, Jungkyu Park, Hyo Shin Kang

**Affiliations:** 1 Psychology, Kyungpook National University, Republic of Korea; 2 Seoul National University Bundang Hospital, Republic of Korea; 3 Neuropsychiatry, Seoul National University Bundang Hospital, Republic of Korea

**Keywords:** Chronotype, depressive symptoms, emotion regulation, suicide attempts, suicidal ideation

## Abstract

**Objective:**

Emotion regulation, while closely linked to depressive symptoms, has seldom been examined together with them in studies of the relationship between chronotype and suicidality. We therefore examined whether chronotype predicts suicidality through the sequential mediation of poor emotion regulation and depressive symptoms. In addition, we examined whether these mediation pathways differ between morning-type and evening-type groups.

**Methods:**

This study included 3109 Korean adults from the general population. Chronotype, depressive symptoms, emotion regulation, and suicidality were assessed using the Composite Scale of Morningness, Self-Rating Depression Scale, Emotion Regulation Skills Questionnaire, and the Suicidality module of the Mini International Neuropsychiatric Interview, respectively.

**Results:**

Chronotype did not have a direct effect on suicidality. Instead, eveningness was indirectly linked to higher suicidality. Specifically, individuals with stronger eveningness tendencies reported poorer emotion regulation, which increased depressive symptoms; depressive symptoms, in turn, predicted suicidal ideation, which emerged as a significant predictor of suicide attempts. Subgroup analyses revealed that the same sequential pathway was significant only among evening-types, but not among morning-types.

**Conclusions:**

Chronotype appears to play a role in suicide risk in the general population. Screening for chronotype and focusing on emotion regulation and depressive symptoms may enhance prevention efforts tailored to chronotype, especially for evening-type individuals.

## Graphical abstract

**Figure:**
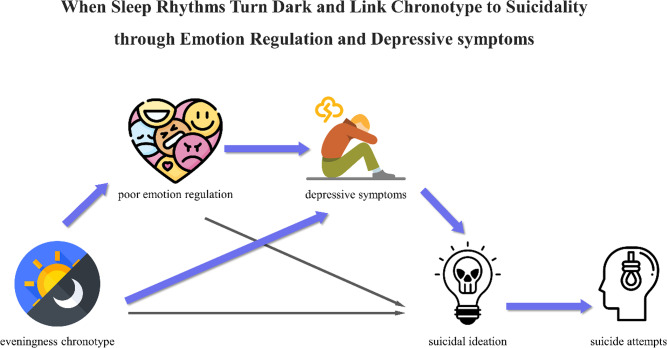

Significant outcomes

- This study extended the understanding of the psychological mechanisms linking chronotype to suicidal ideation and suicide attempts.
- Greater eveningness was associated with poor emotion regulation and depressive symptoms, which sequentially mediated the relationship between chronotype and suicidal ideation, thereby increasing the risk of suicide attempts.
- The sequential mediation was significant only among evening-type individuals, highlighting the importance of screening them in suicide prevention.

Limitations

- Chronotype was measured only through the self-reported scale, which may not accurately capture individuals’ actual behavioural rhythms.
- The cross-sectional design limits causal and temporal interpretations.
- As the sample was not population-based, the findings may have limited generalizability to the broader population.

## Introduction

Circadian typology classifies individuals into three subtypes, known as chronotypes, based on variations in their circadian rhythms, particularly their sleep–wake patterns: morning-types (M-types), evening-types (E-types), and neither-types (N-types) (Horne & Ostberg, 1976; Montaruli *et al*., 2021). Chronotype is influenced by multiple genetic factors and, although it may exhibit modest changes across the lifespan, has been shown in longitudinal studies to represent a relatively stable individual trait (Kalmbach *et al*., 2017; Maukonen *et al*., 2019; Druiven *et al*., 2020). Chronotype is conceptualised as a continuum ranging from morningness to eveningness. Individuals identified as M-types typically go to bed and awaken early, demonstrating peak cognitive and physical functioning during the early hours of the day. In contrast, E-types have later sleep–wake patterns and perform best during the evening (Adan *et al*., 2012). The previous study has demonstrated that approximately 40% of adults fall into distinct chronotypes (i.e., M-types or E-types), while the remaining 60% classified as neither type (i.e., N-types) (Antúnez, 2020; Montaruli *et al*., 2021).

Increasing evidence suggests that chronotype, beyond reflecting behavioural preferences, may function as a transdiagnostic vulnerability factor for various forms of mental illness (Taylor & Hasler, 2018). In particular, an evening chronotype (i.e., eveningness) is associated with higher levels of depressive symptoms (Antypa *et al*., 2016; Bauducco *et al*., 2020) as well as increased challenges in emotion regulation (Azad-Marzabadi & Amiri, 2017). Both factors are known to elevate the risk of suicide. The term suicidal behaviours is commonly used as an umbrella concept encompassing three subcategories: suicide ideation, suicide plan, and suicide attempt (Nock *et al*., 2008). In the present study, following the definitions provided by the US Centers for Disease Control and Prevention, suicide attempts are defined as nonfatal self-directed behaviours performed with the intent to die, whereas suicidal ideation refers to thinking about, considering, or planning suicide (Crosby *et al*., 2011). Within this conceptual distinction, depressive symptoms are widely acknowledged as major psychiatric risk factors for suicide (Bachmann, 2018; Beghi *et al*., 2021), while emotion dysregulation has been consistently associated with suicidal ideation and attempts in both clinical and non-clinical populations (Colmenero-Navarrete *et al*., 2022).

Given these associations, several studies have reported that eveningness is directly associated with higher levels of suicidal ideation and suicide attempts in both adolescents and adults (Gau *et al*., 2007; Selvi *et al*., 2011). However, more recent work suggests that chronotype may indirectly influence suicidality by serving as a distal risk factor that contributes to intermediate vulnerabilities, such as social anxiety, insomnia, mood instability, and emotion dysregulation, which in turn increase the likelihood of psychiatric conditions associated with suicide risk (Magnani *et al*., 2023). Consistent with this perspective, Mendelian randomisation studies accounting for shared genetic liability with sleep inertia suggest that chronotype does not have a direct causal effect on suicide risk (Bruns *et al*., 2024).

Among these psychological vulnerabilities, depressive symptoms have emerged as a particularly robust mediator. For instance, research with university students found that both depressive symptoms and anxiety fully mediated the relationship between eveningness and both suicidal ideation and suicide attempts (Park *et al*., 2018; Nowakowska-Domagała *et al*., 2023). Similar findings have been reported in mixed samples, including individuals with major depressive disorder (MDD) and healthy controls, where depressive symptoms and psychological pain fully mediated the link between chronotype and suicidal ideation (Üzer & Kurtses Gürsoy, 2022). These results underscore internalising symptoms as key pathways through which eveningness may increase susceptibility to suicidality.

In addition to depressive symptoms, difficulties in emotion regulation may represent another important psychological mechanism linking chronotype to suicidality. Emotion regulation involves internal and external processes that monitor, assess, and adjust emotional responses to support goal-directed behaviour (Thompson, 1994). Emotion regulation difficulties have been linked to a wide range of psychopathology (Berking *et al*., 2011; Wirtz *et al*., 2014), particularly depression (Radkovsky *et al*., 2014). Notably, individuals with MDD exhibit greater difficulties in emotion regulation than healthy controls, and these deficits persist even in remitted patients (Visted *et al*., 2018). Neuroimaging studies further suggest that patients with MDD display functional abnormalities in brain regions implicated in emotion regulation (Wu *et al*., 2024), underscoring their role in both the onset and recurrence of depressive symptoms.

These findings suggest that difficulties in emotion regulation may serve as an antecedent mechanism that contributes to the development of depressive symptoms. Moreover, existing evidence indicates that chronotype is associated with emotion regulation tendencies. For instance, M-types tend to employ more adaptive strategies, such as cognitive reappraisal, compared to E-types (Antúnez, 2020). Moreover, depressive symptoms have become more severe among E-types, and this relationship is mediated by reduced use of adaptive emotion regulation strategies (Van den Berg *et al*., 2018). Despite these findings, prior studies that examined the link between chronotype and suicidality have typically focused on depressive symptoms alone, without jointly considering the role of emotion regulation (Park *et al*., 2018; Üzer & Kurtses Gürsoy, 2022).

Furthermore, previous studies have not clearly distinguish between suicidal ideation and suicide attempts (Park *et al*., 2018; Üzer & Kurtses Gürsoy, 2022). From an ideation-to-action perspective, however, such a distinction is critical, as it may help refine suicide theories and inform more targeted clinical interventions (Klonsky *et al*., 2021). Therefore, the present study adopts a dual-pathway model that separately examines ideation and attempts. In addition, consistent with prior research, we classified participants in the top and bottom 20% of chronotype scores into M-types and E-types (Prat & Adan, 2013; Lee *et al*., 2017; Park *et al*., 2018). We then compared these groups to examine whether the pathways to suicidality differ based on chronotype.

Accordingly, the present study aims to examine how poor emotion regulation and depressive symptoms sequentially mediate the relationship between chronotype and suicidality. We hypothesise that eveningness increases the risk of suicidal ideation through poor emotion regulation and heightened depressive symptoms, which in turn increase the likelihood of suicide attempts.

## Material and methods

### Participants

This study was conducted with a non-clinical sample of 3109 individuals. Participants were recruited anonymously through an online survey conducted by a professional survey company between November and December 2024. Individuals with no history of psychiatric treatment were included in the study. Based on the reverse-coded Composite Scale of Morningness (CS) scores, participants in the top 20% of the distribution were classified as the evening-type group (*n* = 645), and those in the bottom 20% were classified as the morning-type group (*n* = 720). No personally identifiable information was collected, ensuring the confidentiality and anonymity of all respondents. Approval for this study was obtained from the Institutional Review Board of Seoul National University Bundang Hospital (B-2205-756-111). Table 1 shows the demographics of samples, including age and sex. Among the full analytic sample, 63.4% were female and the mean age was 38.6. Participants with missing data on any variables used in the analysis were excluded via listwise deletion.

**Table 1.** Demographics (sex and age) of samples

Demographics	*n* (%) or M (SD)
Full sample	
Age	38.6 (12.3)
Sex (1= Female)	1969 (63.4%)
Evening-type	
Age	32.3 (9.5)
Sex (1= Female)	449 (69.7%)
Morning-type	
Age	45.6 (12.0)
Sex (1= Female)	438 (60.9%)

### Measures

#### Composite scale of morningness

The CS is a 13-item self-report questionnaire designed to assess an individual’s sleep–wake patterns and preferred times of activity throughout the day (Smith *et al*., 1989). In the present study, we used the Korean version validated by Kim (1998). Ten items are measured on a 4-point Likert scale (1–4), and three items are measured on a 5-point Likert scale (1–5). Total scores range from 13 to 55. While higher scores usually indicate a greater morningness preference, we reversed the scoring in this study so that higher scores represent a stronger eveningness preference. The Korean version showed good internal consistency (Cronbach’s *α* = 0.81) (Kim, 1998), and in the current study, the scale exhibited excellent reliability (Cronbach’s *α* = 0.88).

#### Self-rating depression scale

The Self-Rating Depression Scale (SDS) is a 20-item self-report measure developed to assess the presence and severity of depressive symptoms (Zung, 1965). The Korean version of the SDS, validated by Lee (1991), was employed in the current study. Participants rated the frequency with which they experienced each symptom over the past week using a 4-point Likert scale (1–4). Total scores range from 20 to 80, with higher scores indicating greater severity of depressive symptoms. The Korean version has demonstrated acceptable internal consistency (Cronbach’s *α* = 0.80) (Lee, 1991) and showed good reliability in the current sample (Cronbach’s *α* = 0.87).

#### Emotion regulation skills questionnaire

The Emotion Regulation Skills Questionnaire (ERSQ) consists of 27 self-report items and is intended to assess a broad spectrum of emotion regulation skills (Berking & Znoj, 2008). The Korean version of the ERSQ (Do *et al*., 2026) was employed in the current study. It comprises nine subscales, each with three items, assessing the following domains: awareness, sensation, clarity, understanding, acceptance, resilience, self-support, tolerance, and modification. Respondents rate each item on a 5-point Likert scale ranging from 0 (‘not at all’) to 4 (‘almost always’) (Grant *et al*., 2018). The Korean version showed excellent composite reliability (*ω* = 0.97) (Do *et al*., 2026). It also demonstrated excellent internal consistency in the present study (Cronbach’s *α* = 0.96).

#### Suicidality

To assess suicidal ideation and suicide attempts, the ‘Suicidality’ module of the Mini International Neuropsychiatric Interview (MINI) was utilised (Sheehan *et al*., 1998; Yoo *et al*., 2006). This module comprises six items that evaluate suicidal ideation, planning, and attempts during the past month, as well as lifetime suicide attempts. Suicidal ideation was measured by summing the weighted scores of Item 1 (‘Think that you would be better off dead or wish you were dead?’) and Item 3 (‘Think about suicide?’). The bivariate correlation between Item 1 and Item 3 was *r* = 0.69, indicating a strong association between the indicators. Suicide attempts were assessed using Item 6 (‘Have you ever made a suicide attempt?’) as a binary indicator of lifetime suicide attempts.

### Statistical analysis

We conducted a mediation path analysis to test the hypothesised model, which posits that chronotype influences suicide attempts through a sequential chain of mediators: emotion regulation, depressive symptoms, and suicidal ideation. Specifically, we examined four indirect pathways: two involving two mediators – (1) chronotype → emotion regulation → suicidal ideation → suicide attempts, and (2) chronotype → depressive symptoms → suicidal ideation → suicide attempts – one involving three mediators in sequence – (3) chronotype → emotion regulation → depressive symptoms → suicidal ideation → suicide attempts, and the other one involving one mediator – (4) chronotype → suicidal ideation → suicide attempts.

Given that the outcome variable, suicide attempts, was binary, a probit link function was used to estimate path coefficients. The model was estimated using the weighted least squares means and variance adjusted (WLSMV) estimator, which is appropriate for models that include categorical variables (Asparouhov & Muthén, 2010; Li, 2021). Age and sex were controlled for in all analyses by including them as covariates.

We first fitted the model using the full sample while controlling for sex and age. This approach allows us to establish the general structural relationships among study variables before examining whether the mediation pathways differ across chronotype groups. In addition, multigroup analyses were conducted to examine whether the mediation effects differed between morning-type and evening-type chronotype groups. To further assess the potential influence of sex and age, additional multigroup analyses were conducted by sex (male vs. female) and by age group (younger vs. older, defined using a ±1 SD split). Model fit was assessed using multiple robust indices, including the RMSEA, CFI, TLI, and SRMR, based on conventional thresholds (e.g., RMSEA < 0.08, CFI > 0.90) recommended by Hu & Bentler (1999) and Kline (2016). The significance of indirect effects was determined using 95% bias-corrected bootstrap confidence intervals, based on 10 000 bootstrap samples. All statistical analyses were performed using Mplus version 8.5 (Muthén & Muthén, 1998–2020).

## Results

The means, standard deviations, and correlations for all study variables in the full sample are presented in Table 2, and descriptive statistics by chronotype groups are provided in Table S1. Among the participants of the full sample, 8.3% (*n* = 257) reported a history of suicide attempts. We first conducted mediation analysis using the full sample and then performed subgroup analysis to examine whether the mediation effects differed across chronotype groups. Moreover, we performed multigroup analyses to further assess the potential influence of control variables, sex and age, with the results presented in Table S2 and Table S3, respectively.

**Table 2.** Means, standard deviations, and correlations of study variables for the full sample

Variables	Means (%)	SD	1	2	3	4	5	6
1. Sex(1= female)	0.64	–						
2. Age	38.22	12.13	−0.053**					
3. Chronotype	21.19	7.41	0.106***	−0.384***				
4. Regulation	61.79	18.76	−0.011	−0.087***	−0.120***			
5. Depressive Symptoms	40.88	8.76	0.131***	−0.110***	0.337***	−0.311***		
6. Suicidal Ideation	0.78	2.11	0.087***	−0.049*	0.133***	−0.128***	0.387***	
7. Suicide Attempts	0.08	–	0.099*	0.042	0.094**	−0.119***	0.272***	0.280***

*Note*: Correlations between binary (1. sex and 7. suicidal attempt) and continuous variables were computed as point-biserial correlations, and correlation between two binary variables was computed as phi coefficient.

*p < 0.05. **p < 0.01. ***p < 0.001.

**Figure 1.**
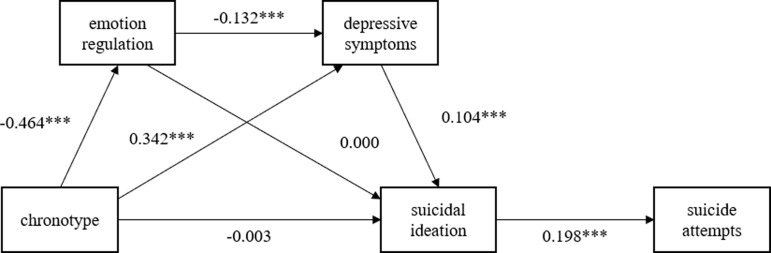
Estimated model for full sample. *Note*: The mediation analysis on the relationship between chronotype and suicide attempts; Values shown are unstandardised path coefficients; *p < 0.001.

The model demonstrated an acceptable fit to the data (χ^2^(3) = 39.079, *p* < 0.001; CFI = 0.967; TLI = 0.803; SRMR = 0.062; RMSEA = 0.062, 90% CI [0.046, 0.080]). Figure 1 presents the path diagram along with the estimated path coefficients. The results show that eveningness (high score in chronotype items) is negatively associated with emotion regulation (*β* = −0.464, *p* < 0.001), while it is positively associated with depressive symptoms (*β* = 0.342, *p* < 0.001). Moreover, there was no significant direct effect of chronotype on suicidal ideation, suggesting that being an evening-type does not directly increase the likelihood of experiencing suicidal ideation (*β* = 0.003, *ns*). Emotion regulation is negatively associated with depressive symptoms (*β* = −0.132, *p* < 0.001), while depressive symptoms are positively associated with suicidal ideation (*β* = 0.104, *p* < 0.001). The path from suicidal ideation to suicide attempts was significant and positive (*β* = 0.198, *p* < 0.001), indicating that greater suicidal ideation corresponds to a higher underlying likelihood of engaging in suicide attempts.

Table 3 summarises four indirect effects along with their 95% confidence intervals for the full sample and chronotype groups. The indirect effect from chronotype to suicide attempts through the serial mediation of depressive symptoms and suicidal ideation is positive and significant (*B* = 0.007, *p* < 0.001, 95% CI [0.005, 0.010]). Similarly, the indirect effect through emotion regulation, depressive symptoms, and suicidal ideation is also positive and significant (*B* = 0.001, *p* < 0.001, 95% CI [0.001, 0.002]). These findings suggest that emotion regulation and depressive symptoms are important factors connecting chronotype and suicide attempts via suicidal ideation; however, emotion regulation alone does not function as a sole mediator. Additionally, the indirect effect mediated only by suicidal ideation was not significant (95% CI [−0.003, 0.002]), indicating that belonging to the evening-type groups does not lead to suicide attempts through suicidal ideation alone, highlighting the crucial roles of emotion regulation and depressive symptoms as mediators.

**Table 3.** Estimates, standard errors and 95% confidence intervals for path coefficients and indirect effects

Indirect effects	Estimate	SE	95%CI	
			Lower	Upper
Full sample				
X→Y→Z	−0.001	0.001	−0.003	0.002
X→M1→Y→Z	0.000	0.000	0.000	0.000
X→M2→Y→Z	0.007***	0.001	0.005	0.010
X→M1→M2→Y→Z	0.001***	0.000	0.001	0.002
Evening-type group				
X→Y→Z	0.006	0.008	−0.009	0.024
X→M1→Y→Z	0.001	0.001	−0.001	0.004
X→M2→Y→Z	0.014**	0.005	0.007	0.025
X→M1→M2→Y→Z	0.003*	0.001	0.001	0.007
Morning-type group				
X→Y→Z	−0.006	0.005	−0.018	0.002
X→M1→Y→Z	0.000	0.001	−0.001	0.002
X→M2→Y→Z	0.004	0.003	0.001	0.012
X→M1→M2→Y→Z	0.001	0.001	0.000	0.004

*Note*: X = Chronotype, M1 = Emotion Regulation, M2 = Depressive Symptom, Y = Suicidal Ideation, Z = Suicide Attempts.

*p < 0.05. **p < 0.01. ***p < 0.001.

**Figure 2.**
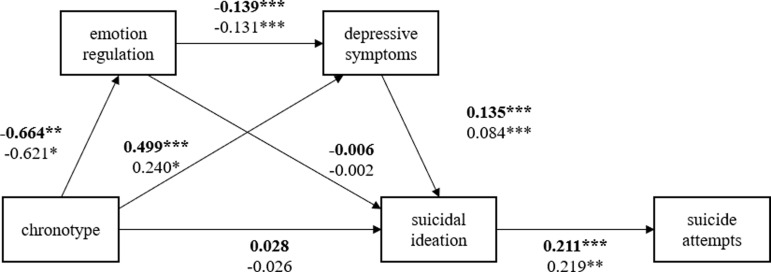
Estimated model by chronotype groups. *Note*: Boldfaced coefficients represent the evening-type group, and the non-bolded coefficients represent the morning-type group; *p < 0.001.

Subsequently, a multigroup analysis was conducted to examine whether these mediation pathways differed between the morning-type and evening-type groups. The fit indices for the multigroup model were acceptable (*χ^2^* (6) = 19.888, *p* = 0.003; CFI = 0.961; TLI = 0.766; SRMR = 0.086; RMSEA = 0.058, 90% CI [0.031, 0.088]). Figure 2 displays the path diagram and estimated path coefficients for each group. The Wald test for comparing the path coefficients indicated that there were no significant differences between the groups. In the evening-type group, the indirect effect of chronotype on suicide attempts via emotion regulation, depressive symptoms, and suicidal ideation was significant (*B* = 0.003, *p* = 0.050, 95% CI [0.001, 0.006]), while this effect was not significant in the morning-type group (*B* = 0.001, *p* = 0.112, 95% CI [0.000, 0.004]). This pattern suggests a stronger mediation effect in the evening-type group. In addition, the estimate of serial indirect effect – chronotype→ depressive symptoms →suicidal ideation→ suicide attempts – was greater in the evening-type group (*B* = 0.014, *p* = 0.004, 95% CI [0.007, 0.025]) relative to the morning-type group (*B* = 0.004, *p* = 0.114, 95% CI [0.001, 0.012]), indicating that this pathway has a stronger positive influence among individuals with an evening-type chronotype.

## Discussion

This study investigated whether poor emotion regulation and depressive symptoms sequentially mediated the relationship between chronotype and suicidality in a general population sample. It also examined whether these mediation pathways differed between M-types and E-types. In the entire sample, path analysis revealed that chronotype indirectly influenced suicide attempts through a sequential pathway involving poor emotion regulation, depressive symptoms, and suicidal ideation; the total indirect effect was statistically significant. In subgroup analyses, the indirect pathway from chronotype to suicide attempts via depressive symptoms and suicidal ideation was significant in the E-types but not in the M-types.

Consistent with previous literature, the present study identified significant associations among chronotype, emotion regulation, depressive symptoms, and suicidality. Prior research has demonstrated that chronotype is linked to both suicidality (Rumble *et al*., 2018, 2020; Üzer *et al*., 2024) and depressive symptoms (Kivelä *et al.*, 2018; Seo *et al*., 2022; Kang *et al*., 2024). Furthermore, emotion regulation difficulties have been consistently associated with depressive symptoms and conceptualised as a transdiagnostic mechanism underlying various psychological disorders (Sloan *et al*., 2017; Kraft *et al*., 2023). Despite these well-established associations, few studies have tested an integrated model that simultaneously includes chronotype, emotion regulation, depressive symptoms, and suicidality. By proposing and testing a sequential mediation model, the present study builds upon existing evidence and contributes to a more integrative understanding of the psychological pathways linking chronotype to suicidal behaviour.

This study also demonstrated that chronotype influences suicidality not directly, but indirectly through a full mediation pathway. Specifically, depressive symptoms emerged as a significant mediator, fully accounting for the relationship between chronotype and suicidality. This finding aligns with previous studies indicating that individuals with evening-type tendencies are more likely to experience elevated depressive symptoms, which in turn increase suicidality (Park *et al*., 2018; Mokros *et al*., 2021; Üzer & Kurtses Gürsoy, 2022; Nowakowska-Domagała *et al*., 2023). Whereas depressive symptoms were found to play a significant mediating role in the association between chronotype and suicidality, emotion regulation did not independently mediate the relationship between these variables. However, despite the non-significant indirect effect of emotion regulation alone, the sequential indirect effect involving both emotion regulation and depressive symptoms in the association between chronotype and suicidality was significant. These findings suggest that emotion regulation may not be directly associated with suicidality, but may be indirectly related to suicidality through its association with depressive symptoms.

These patterns underscore the need to understand how chronotype shapes emotional processing at both psychological and neural levels. Neuroimaging evidence suggests that structural and functional brain differences may partly explain the link between chronotype, emotion regulation, and depressive symptoms. Specifically, a structural MRI study revealed that individuals with an evening chronotype exhibited localised atrophy in the subiculum region of the right hippocampus (Horne & Norbury, 2018a). Functionally, evening-type individuals also showed heightened amygdala reactivity to negative emotional facial expressions and reduced connectivity between the dorsal anterior cingulate cortex (dACC) and the amygdala, according to a related fMRI study (Horne & Norbury, 2018b). These brain regions – the hippocampus, amygdala, and dACC – are critically involved in emotion regulation, and abnormalities in their structure or connectivity have been linked to depressive symptoms (Phillips *et al*., 2008; Kaiser *et al*., 2015; Park *et al*., 2019; Zhu *et al*., 2019; Berboth & Morawetz, 2021). Consistent with these neuroimaging findings, a recent large-scale pattern-learning analysis identified spatial convergence between chronotype and key limbic regions implicated in emotional processing (Zhou *et al*., 2025).

In addition to neurobiological factors, the link between chronotype and depressive symptoms can be understood through psychological mechanisms. Clinical studies have shown that circadian rhythm disruptions are associated with impairments in emotion regulation, such as emotional awareness, clarity, and acceptance, which in turn mediate the relationship between depressive symptoms and suicidality (Palagini *et al*., 2019; Palagini *et al*., 2022). External factors such as school schedules, shift work, and jet lag can disrupt the alignment between one’s daily routines and the internal biological clock, referred to as circadian rhythm disruption or social jet lag (SJL) (Zou *et al*., 2022). This form of circadian misalignment tends to be more pronounced in individuals with a late chronotype (Roenneberg *et al*., 2019). The present findings may offer preliminary evidence that similar mechanisms could be relevant in general populations. Moreover, individuals with an evening chronotype may be more vulnerable to depressive symptoms due to the specific emotion regulation strategies they tend to use. They are less likely to engage in cognitive reappraisal and more likely to rely on expressive suppression and self-blame, which may contribute to depressive symptoms and increase vulnerability to psychiatric disorders (Watts & Norbury, 2017; Van den Berg *et al*., 2018).

The present findings underscore the importance of distinguishing between suicidal ideation and suicide attempts, particularly in the context of chronotype. While not all individuals who experience suicidal ideation proceed to suicide attempts, understanding the factors that influence this progression is essential (Bayliss *et al*., 2022). The Ideation-to-Action Framework offers one explanation, proposing that the capability for suicide is a key factor enabling the transition from ideation to action (Keefner & Stenvig, 2020). In this framework, pain and hopelessness have been identified as major contributors to suicidal desire (Klonsky *et al*., 2018), and individuals with an evening chronotype are more likely to experience heightened levels of these vulnerabilities. Empirical studies have shown that psychological pain mediates the relationship between chronotype and suicidality in both clinical and non-clinical samples (Üzer & Kurtses Gürsoy, 2022; Üzer *et al*., 2024), while hopelessness mediates the association between chronotype and depressive symptoms (Üzer & Yücens, 2020). These findings suggest that chronotype may function as a distal vulnerability factor in suicidality, increasing the risk for both ideation and attempts through specific psychological mechanisms.

In this study, the indirect effect of chronotype on suicide attempts via depressive symptoms and suicidal ideation was significant only among E-types, while the same pathway was non-significant among M-types. Although the group differences were only marginally significant and did not meet the conventional threshold, this pattern may reflect heightened psychological vulnerability in E-types. Prior studies have shown that E-types tend to exhibit greater psychological risk factors, such as higher levels of rumination (Antypa *et al*., 2017) and lower behavioural activation system, and reduced positive affect, which may increase the risk of depressive symptoms (Hasler *et al*., 2010). In line with these findings, the E-types in our sample exhibited higher levels of depressive symptoms, which may partially account for the observed group-specific pathway. By contrast, morningness has been proposed as a potential protective factor against depressive symptoms and suicidality (Gaspar-Barba *et al*., 2009; Park *et al*., 2018). In particular, evidence from Mendelian randomisation studies suggests that genetically predicted morningness is causally associated with a lower risk of MDD (Daghlas *et al*., 2021; Sun *et al*., 2022; Qiu-Qiang *et al*., 2024).

These findings emphasise the potential utility of chronotype-informed approaches to suicide prevention, particularly for individuals with an evening preference. Given that chronotype can be assessed easily and non-invasively in the general population, it may represent a useful marker for identifying individuals who report higher levels of depressive symptoms and suicidal ideation at the population level. Moreover, the observed associations involving emotion regulation difficulties and depressive symptoms point to psychological processes that may warrant further attention as potential targets for early preventive efforts.

Several limitations of the present study should be acknowledged, along with directions for future research. First, chronotype was assessed solely through the CS, a self-report measure that captures preferred timing of daily activities. However, this may not fully reflect individuals’ actual behavioural rhythms (Bauducco *et al*., 2020). Future studies should incorporate both subjective (e.g., CS) and behavioural measures (e.g., actigraphy, sleep diaries) to enhance the precision and validity of chronotype assessment. Second, the cross-sectional design limits the ability to draw temporal inferences and constrains causal interpretation. In particular, suicide attempts were assessed using a lifetime indicator, whereas the mediators were measured over the past week or month. Because time-specific or repeated assessments of suicide attempts were not available, the temporal ordering implied by the mediation model could not be directly tested. Thus, caution is warranted when interpreting the directionality and temporal sequence of the observed associations. Given that suicide capability can fluctuate over time (Spangenberg *et al*., 2019), longitudinal research with multiple time points is necessary to better understand the progression from suicidal ideation to suicide attempts. Third, the study sample was not population-based and therefore may not be fully representative of the general population, which limits the generalizability of the findings.

In conclusion, the present study found that poor emotion regulation and depressive symptoms were significantly associated with the link between chronotype and both suicidal ideation and suicide attempts in a general population sample. By extending prior research predominantly conducted in clinical samples, this study highlights the psychological vulnerability associated with eveningness even among non-clinical individuals. These findings underscore the relevance of emotion regulation difficulties and depressive symptoms in relation to suicide risk, particularly in evening-type individuals. Moreover, these findings highlight the importance of accounting for individual differences in circadian preference, as they may be indirectly associated with the transition from suicidal ideation to suicide attempts through their effects on emotional and affective processes.

## Supporting information

10.1017/neu.2026.10077.sm001 Choo et al. supplementary material Choo et al. supplementary material

## Supplementary material

The supplementary material for this article can be found at https://doi.org/10.1017/neu.2026.10077.
