# Supplementary material for: Emotion regulation and depressive symptoms mediate the association between chronotype and suicidality
Source: Acta Neuropsychiatr. 2026 Apr 10;38:e32. doi: 10.1017/neu.2026.10077 (PMC13202391; doi:10.1017/neu.2026.10077)
Supplement: Choo et al. supplementary material [file S0924270826100775sup001.docx]

Table S1. Means, standard deviations, and Correlations of Study Variables (For the samples by chronotype groups)

| **Variables** | **Means**  **(%)** | **SD** | **1** | **2** | **3** | **4** | **5** | **6** |
| --- | --- | --- | --- | --- | --- | --- | --- | --- |
| **Eveningness group** | | | | | | | | |
| 1. sex(1=female) | 0.70 | - |  |  |  |  |  |  |
| 2. age | 32.33 | 9.53 | -0.067 |  |  |  |  |  |
| 3. chronotype | 31.66 | 3.13 | 0.062 | -0.122^**^ |  |  |  |  |
| 4. regulation | 57.85 | 19.07 | -0.026 | -0.108^**^ | -0.095^**^ |  |  |  |
| 5. depressive  symptoms | 45.35 | 8.44 | 0.074 | 0.049 | 0.211^***^ | -0.327^***^ |  |  |
| 6. suicidal  ideation | 1.27 | 2.56 | 0.143^*^ | 0.059 | 0.120^**^ | -0.187^***^ | 0.419^***^ |  |
| 7. suicide  attempts | 0.10 | - | 0.077 | 0.061 | 0.110 | -0.138^*^ | 0.306^***^ | 0.361^***^ |
| **Morningness group** | | | | | | | | |
| 1. sex(1=female) | 0.61 | - |  |  |  |  |  |  |
| 2. age | 45.64 | 12.02 | -0.136^**^ |  |  |  |  |  |
| 3. chronotype | 11.47 | 3.10 | 0.074 | -0.149^***^ |  |  |  |  |
| 4. regulation | 63.85 | 19.34 | 0.001 | -0.141^***^ | -0.075^*^ |  |  |  |
| 5. depressive  symptom | 36.97 | 8.35 | 0.151^**^ | -0.053 | 0.130^***^ | -0.291^***^ |  |  |
| 6. suicidal  ideation | 0.55 | 1.80 | 0.023 | -0.097^*^ | 0.014 | -0.111^**^ | 0.341^***^ |  |
| 7. suicide  attempts | 0.07 | - | 0.232^*^ | 0.029 | 0.042 | -0.095 | 0.299^***^ | 0.241^***^ |

Note. Correlations between binary (1. sex and 7. suicide attempts) and continuous variables were computed as point-biserial correlations, and correlation between two binary variables was computed as phi coefficient.

^*^*p*<.05. ^**^*p*<.01. ^***^*p* <.001.

Table S2. Estimates, standard errors and 95% confidence intervals for indirect effects in the multi-group analysis by sex

| Indirect effects | Male | | | | Female | | | |
| --- | --- | --- | --- | --- | --- | --- | --- | --- |
|  | Estimate | SE | 95%CI |  | Estimate | SE | 95%CI |  |
|  |  |  | Lower | Upper |  |  | Lower | Upper |
| X→Y→Z | -0.002 | 0.002 | -0.007 | 0.003 | 0.000 | 0.002 | -0.003 | 0.003 |
| X→M1→Y→Z | 0.000 | 0.000 | -0.001 | 0.001 | 0.000 | 0.000 | -0.001 | 0.001 |
| X→M2→Y→Z | 0.008^***^ | 0.002 | 0.004 | 0.012 | 0.007^***^ | 0.001 | 0.004 | 0.010 |
| X→M1→M2→Y→Z | 0.001^*^ | 0.000 | 0.000 | 0.002 | 0.001^***^ | 0.000 | 0.001 | 0.002 |

Note. X = Chronotype, M1 = Emotion Regulation, M2 = Depressive Symptoms, Y = Suicidal Ideation, Z = Suicide Attempts.

^*^*p*<.05. ^***^*p* <.001.

Table S3. Estimates, standard errors and 95% confidence intervals for indirect effects in the multi-group analysis by age group (±1 SD)

| Indirect effects | Younger group | | | | Older group | | | |
| --- | --- | --- | --- | --- | --- | --- | --- | --- |
|  | Estimate | SE | 95%CI |  | Estimate | SE | 95%CI |  |
|  |  |  | Lower | Upper |  |  | Lower | Upper |
| X→Y→Z | -0.005 | 0.004 | -0.016 | 0.002 | -0.001 | 0.002 | -0.005 | 0.004 |
| X→M1→Y→Z | 0.001 | 0.001 | 0.000 | 0.003 | 0.000 | 0.000 | -0.001 | 0.000 |
| X→M2→Y→Z | 0.006^**^ | 0.002 | 0.003 | 0.012 | 0.007^**^ | 0.003 | 0.003 | 0.012 |
| X→M1→M2→Y→Z | 0.002^*^ | 0.001 | 0.000 | 0.004 | 0.000 | 0.000 | 0.000 | 0.001 |

Note. X = Chronotype, M1 = Emotion Regulation, M2 = Depressive Symptoms, Y = Suicidal Ideation, Z = Suicide Attempts; Younger group = participants with age ≤ (mean − 1 SD); Older group = participants with age ≥ (mean + 1 SD); Participants within ±1 SD of the mean age were excluded from this multi-group analysis (N = 1,188)

^*^*p*<.05. ^**^*p*<.01.
